# Technology-assisted adaptive recruitment strategy for a large nation-wide COVID-19 vaccine immunogenicity study in Brunei

**DOI:** 10.3389/fpubh.2022.983571

**Published:** 2022-09-12

**Authors:** Chin Yee Shim, Si Yee Chan, Yuan Wei, Hazim Ghani, Liyana Ahmad, Hanisah Sharif, Mohammad Fathi Alikhan, Saifuddien Haji Bagol, Surita Taib, Chee Wah Tan, Xin Mei Ong, Lin-Fa Wang, Yan Wang, An Qi Liu, Hong Shen Lim, Justin Wong, Lin Naing, Anne Catherine Cunningham

**Affiliations:** ^1^EVYD Research Pte Ltd., A Subsidiary of EVYD Technology Limited, Singapore, Singapore; ^2^PAPRSB Institute of Health Sciences, Universiti Brunei Darussalam, Bandar Seri Begawan, Brunei; ^3^Disease Control Division, Ministry of Health, Bandar Seri Begawan, Brunei; ^4^Department of Laboratory Services, Ministry of Health, Bandar Seri Begawan, Brunei; ^5^Programme in Emerging Infectious Diseases, Duke-NUS Medical School, Singapore, Singapore

**Keywords:** digital technology, adaptive recruitment, data integration, electronic medical records, contact tracing, neutralising antibodies (NAB), COVID-19 vaccine

## Abstract

A national study was conducted in Brunei to assess and compare the immunogenicity of the various brands of COVID-19 vaccines administered to the population as part of the National COVID-19 Vaccination Programme. Most of the population have had received at least 2 doses of BBIBP-CorV, AZD1222 or MRNA-1273 vaccines. Neutralising antibodies against SARS-CoV-2 induced by these vaccines will be analysed to infer population-level immune protection against COVID-19. During the 5-week recruitment period, 24,260 eligible individuals were invited to the study *via* SMS, out of which 2,712 participants were enrolled into the study. This paper describes the novel adaptive strategy used to recruit the study participants. Digital technology was leveraged to perform targeted online recruitment to circumvent the limitations of traditional recruitment methods. Technology also enabled stratified random selection of these eligible individuals who were stratified based on age, gender and vaccine brand. Data was extracted from the electronic health records, the national mobile health application and a third-party survey platform and integrated into a dedicated research platform called EVYDResearch. The instant availability and access to up-to-date data on EVYDResearch enabled the study team to meet weekly and adopt an adaptive recruitment strategy informed by behavioural science, where interventions could be quickly implemented to improve response rates. Some examples of these include incorporating nudge messaging into SMS invitations, involving the Minister of Health to make press announcements on this study, media coverage, setting up an enquiries hotline and reaching out to foreign language speaking expatriates of a local multinational company to participate in this study. Data integration from various data sources, real time information sharing and a strong teamwork led to good outcomes adaptable to the progress of recruitment, compared to the more time-consuming and static traditional recruitment methods.

## Introduction

Recruiting eligible participants into research studies is challenging (1, 2). Recruiting study participants using traditional methods is a labour and resource intensive process. There are high financial costs involved in obtaining sufficient resources to run recruitment sites and execute the logistics, such as conducting on-site recruitment and interviews, data entry and various administrative duties (3).

The biggest bottleneck prior to starting any health-related study is identifying eligible study participants (4). Electronic health record (EHR) systems in many modern healthcare institutions are rich sources of real-world data (RWD) and can be leveraged to automatically generate a list of patients who fulfil the inclusion and exclusion criteria (5, 6). Having access to data from EHR and other different sources required for clinical studies gives researchers a preliminary expectation of the recruitment rate, and facilitates planning for recruitment (7).

Another important consideration when selecting study participants is to ensure they are representative of the population. Probability sampling eliminates selection bias as any individual in the target population has an equal chance of being selected into the study (8). In clinical research, it may not always be feasible to conduct random sampling in practice due to the inability to identify eligible study participants beforehand, as well as resource and time limitations. Researchers often resort to convenience sampling due to cost or difficulties in recruitment at the expense of statistical bias and lack of representation (9). Stratified random sampling is a more rigorous type of probability sampling and allows for comparison between strata to draw meaningful conclusions. Access to data from different sources like EHR and census has enabled the use of stratified random sampling by identifying eligible sampling population efficiently beforehand (10).

Study teams need to successfully execute recruitment strategies. Success can be monitored by evaluating the recruitment process and performance (11). Technology can produce real-time data which gives prompt insights into the current recruitment progress. Data-driven insights must be communicated regularly with the study teams (12) so that feedback for improvements can be given (13), and prompt, targeted adjustments of recruitment strategies can be made to improve recruitment outcomes. Synchronising the recruitment planning reduces the burden of labour and resource, which leads to improved response and streamlined data collection (12).

The aim of this paper is to present the method and recruitment strategy of a technology assisted adaptive recruitment strategy for a national level study to infer the current level of immune protection against COVID-19 at the population level in Brunei Darussalam. Firstly, participants' data were integrated from the national EHR database and other sources and then scanned for eligibility. Eligible individuals were randomly sampled and invited to the study. The live response rates were shared with stakeholders weekly to enable adaptive recruitment. Data on the confirmed participants were imported into a secure and collaborative research platform for data collection and analysis.

### Background

Vaccination is effective in preventing severe illnesses and death from COVID-19 (14–16). However, different vaccine brands can elicit varying degrees of immune responses (17–20). Their effectiveness can be assessed by measuring the levels of specific antibody in the serum which can be used as a correlate of COVID-19 protection post-vaccination (21). Anti-SARS-CoV-2 antibodies can persist for several months after vaccination, but have been shown to wane over time (22, 23). Particularly, high levels of blocking antibodies called neutralising antibodies (NAb) have been correlated with survival (24, 25) and appear to be the best measure of vaccine efficacy (26).

It is of great interest to monitor immune responses to the various vaccine preparations in populations to evaluate the effectiveness of these programmes (27). Limited data exist on the comparative immunogenicity of different COVID-19 vaccines in Southeast Asian populations. In Brunei, different vaccine types which were distributed for the primary immunisation series to adults included BBIBP-CorV (whole inactivated virus), AZD1222 (viral vector) and mRNA-1273 (mRNA) (28). This was followed by a booster dose of either mRNA-1273 or BNT1626b (mRNA) administered at least 3 months following completion of the primary series (29). The technology assisted adaptive recruitment strategy was implemented in a cross-sectional large nation-wide COVID-19 vaccine immunogenicity study in Brunei. This national study aims to obtain 3,000 blood samples from eligible individuals who received at least two doses of BBIBP-CorV, AZD1222 or mRNA-1273 COVID-19 vaccines (30, 31). Their level of NAb specific to SARS-CoV-2 induced by these vaccines will be analysed using a surrogate virus neutralisation test (32).

## Materials and equipment

### Data source

To identify the eligible population for sampling, data pertinent to study inclusion and exclusion criteria were obtained from different sources. These variables are described below and also listed in Supplementary Table 1 together with their sources. Refer to the Methods section for definitions of the inclusion and exclusion criteria.

Most patient data were obtained from the Brunei Darussalam Health Information System (Bru-HIMS) and BruHealth (Supplementary Table 1). Bru-HIMS is the national electronic health record (EHR) system used in Brunei which has more than 95% of the population's primary and secondary healthcare data across the entire government health network (33). In addition to this, a national mobile health application developed and managed by EVYD Technology Limited (EVYD) called BruHealth was launched by the Ministry of Health, Brunei Darussalam (MOH), in May 2020 to provide a virtual platform to control the spread of COVID-19 in Brunei *via* digital contact tracing and a QR code premise check-in (34, 35). The BruHealth backend framework integrates with Bru-HIMS, allowing for communication among the subsystems. This data aggregation allows BruHealth to be further developed into a population health management app for Bruneians with more extensive functionalities, including health self-assessment during the pandemic, self-quarantine management, access to personal health records, activity trace map of COVID-19 positive patients, map of medical resources, online appointments for COVID-19 vaccinations and medical consultations, plus health management plans like customised exercise programmes (35).

Patient information relating to personal COVID-19 vaccination records were obtained from BruHealth. Other data pertinent to the study inclusion and exclusion criteria, socio-demographics variables and co-morbidities (extracted based on the International Statistical Classification of Diseases and Related Health Problems 10th Revision [ICD-10]) were obtained from both Bru-HIMS and Qualtrics online survey software (36). Data from these datasets were then incorporated into a proprietary research platform developed by EVYD called EVYDResearch. District of residence and immunosuppressive drugs were collected at recruitment site and directly entered into EVYDResearch by data loggers.

Based on the predicted response rate and current capacity, a subset of identified eligible individuals from the above data sources were invited into the study by short messaging service (SMS). This included a link to a questionnaire to indicate their agreement to participate in the study. The questionnaire also contains a link to the Patient Information Sheet in English and Malay (37, 38). If they agree to participate, their data will be imported into the research platform. If they do not wish to participate, their data will not be included in the study. All participants were registered on arrival at the study site to verify details and ensure inclusion and exclusion criteria were met. Participants were briefed about the study and given opportunities to ask clarifying questions before physically signing a consent form before blood sampling. Once informed consent was given, the phlebotomist drew 5mL of blood samples from each participant *via* venepuncture (31).

### Data management

EYVDResearch is engaged as the data management platform for this study (39). It is a web-based application on a secure cloud that allows patients' RWD ingestion from multiple sources, such as Bru-HIMS, BruHealth and Qualtrics survey platform. These datasets have undergone a standard data engineering process called extract, transform, load (ETL).

The aim of ETL is to obtain a complete view of participants' RWD which are normally disparate and housed in different sources which can have differing formats. The ETL process involves extraction of all data from the various sources, transforming the data into a cleaner and more readable format and finally hosting the clean data in a secure, on-premise centralised data operating platform called EVYDENCE. Data quality checks automatically occur after the data has loaded into the EVYDENCE database. These involve checking for empty fields, compliance to field types, field lengths and standardised key words, consistency of linked data and temporal logic. Any errors in the data will be flagged for manual checking. The data will then be processed into high-quality structured data. The necessary data from EVYDENCE can be extracted to EVYDResearch. The high-quality standardised and structuralised data facilitates effective search queries and statistical analyses.

Centralising all RWD onto EVYDResearch means better data management for collaborators to perform complex patient searches and track research milestones, including current recruitment progress. There are dashboards for data visualisation and modules to conduct customisable search queries and statistical analysis. The data can also be exported for in-depth analysis on dedicated statistical software. There is also a track data versioning for auditing purposes.

In this study, MOH retains data ownership of all patients' RWD and holds the rights to grant data access to authorised personnel for the purposes of conducting the research. EVYDResearch follows the data security and privacy requirements of MOH. There is a permission management platform for an independent administrator to distribute or withdraw the data permission for the necessary users *via* the individual metadata settings. Users can only read and/or amend the necessary fields as per the authorisation given by MOH depending on their roles.

Due to the sensitivity of the data, non-disclosure agreements were signed prior to commencing the research. As data on EVYDResearch is hosted on the MOH server, external users are required to obtain consent from MOH to access the virtual private network and EVYDResearch. Participants' identifiable details are confidential and were not disclosed to the study teams on EVYDResearch. Verification of participants' information was only conducted by data loggers at the recruitment site. Each study participant on EVYDResearch was deidentified and assigned a patient ID and a cPass ID which were used as their identifiers (Supplementary Table 1). The cPass ID would only be assigned if they have given their blood sample.

### Ethics approval

This study is approved by the Medical and Ethical Research Committee (MHREC) of the Ministry of Health, Brunei Darussalam (Reference No: MHREC/MOH/2021/14(1) dated 18th November 2021.

## Methods

### Recruitment sampling

The sampling population included all eligible patients who meet the inclusion and exclusion criteria as defined in Table 1. Individuals who met the inclusion criteria were fully vaccinated with either BBIBP-CorV, AZD1222 or mRNA-1273 COVID-19 vaccines for at least 15 days but no more than 45 days (3 to 6 weeks' interval), and are at least 18 years of age. Exclusion criteria included a history of COVID-19 or overseas travel between receiving the first dose of vaccine and blood sample collection (Table 1).

**Table 1:** Inclusion and exclusion criteria of study participants.

**Factor**	**Criteria**	**Further criteria**
COVID-19 vaccine brand	First 2 doses of AZD1222D1222 ± 3rd dose of mRNA-1273 or BNT162b2	The interval between the first 2 doses are 35 days to 63 days inclusive.
	First 2 doses of mRNA-1273 ± 3rd dose of mRNA-1273 or BNT162b2	The interval between the first 2 doses are 21 days to 56 days inclusive.
	First 2 doses of BBIBP-CorV ± 3rd dose of mRNA-1273, BNT162b2 or BBIBP-CorV	The interval between the first 2 doses are 21 days to 35 days inclusive.
Age	18 years old and above	
Gender	Female	
	Male	
Time after receipt of second or third vaccine dose (“last dose”)	15 days to 45 days (inclusive)	
COVID-19 infection	No medical history of COVID-19 infection	
Travel history	No travel history after receipt of first vaccine dose	

Socio-demographic variables including age, sex, ethnicity and district of residence are included in the study. Co-morbidities like diabetes mellitus type 2, chronic kidney diseases, cardiovascular diseases, ischaemic heart diseases, cancers and immunosuppressive drugs were also included in the study.

The sample size for each vaccination brand is 1,000. As individual immunity may vary between gender, age, vaccine brands and time elapsed after receipt of vaccine, the study participants were randomly selected and stratified by vaccine brands, gender, age groups and by number of weeks after receipt of the last vaccine dose. The targeted sample size would be the same and equally distributed across all strata (Supplementary Tables 2, 3). The stratified random sampling was performed using a pseudorandom number generator called Mersenne Twister that was run on Python version 2.7.15 (40). This involved assigning random numbers to all eligible individuals and letting the algorithm randomly select the required number of individuals to be invited from each stratum.

### Recruitment workflow

The recruitment was staged into 5 weekly batches. Each batch required a fortnight of careful planning and prompt executions by different stakeholders as detailed below and in Supplementary Figure 1.

The recruitment methodology was designed based on the underlying principle of having equal sample sizes across all strata (as shown in Supplementary Tables 2, 3). Ideally, each batch would consist of 750 consenting participants from the same post-vaccination week (see Supplementary Table 4) and all strata would have equal representation (see Supplementary Table 5).

Bearing the above principle in mind, the recruitment strategy would be adaptive to the progress of recruitment, and oversample or undersample the relevant strata as appropriate. For this reason, the final batch would serve as a buffer in case of shortfalls in recruitment numbers. Details of the adaptations to the recruitment strategy are elaborated in sections batch 1 to batch 5. Individuals who agreed to participate would be sampled once only for this study. Individuals who did not consent to join or did not respond were not contacted again to join this study another time.

The maximum capacity of the recruitment centre for each study week (depending on manpower and venue availability) restricted the maximum number of blood samples which can be obtained in each batch. Up to seven hourly slots were available daily, and each phlebotomist was estimated to withdraw five blood samples per hour. The expected response rate was decided during weekly meetings guided by the previous batches' response rates to determine the maximum number of invitations which can be sent out, and was calculated as follows:


$$\begin{array}{l} No.\;of\;invitations\;to\;send\;out=\frac{Maximum\;capacity}{Expected\;response\;rate} \end{array}$$

In order to prepare the resources on-site, participants were asked to register their agreement to participate in the study on a survey form hosted on a third-party survey platform called Qualtrics. Invitation SMSs with the survey link were sent to selected potential study participants. A link to the patient information sheet was also provided in the survey link for information regarding the study. Refer to Table 2 for the SMS templates. Participants were given 3 days to respond to the Qualtrics survey, and a reminder SMS was sent the day before Qualtrics closure. Qualtrics responses were reviewed to estimate the probable attendance at the recruitment centre and optimised resource availability.

**Table 2:** Template of the SMS invitations and reminders sent to invited eligible individuals to participate in the study. Nudge messaging was incorporated into SMSes from Batch 3 onwards.

	**Invitation SMS**	**Reminder SMS**
Original text	[MOH] IC:01234567 You are invited to a vaccine research study on dd/mm/yy at 08:00 AM-09:00 AM. Please respond at [survey link] by Wednesday, 5:00 PM. This invitation is personalised for you.	[MOH] IC:01234567 Reminder to respond to your vaccine research study invitation at [survey link] by Wednesday, 05:00 PM. Please disregard this message if you have already responded.
Nudge messaging amendments from Batch 3 onwards	No change	[MOH] IC:01234567 **An antibody test is reserved for you next week. Please confirm your attendance** at [survey link] by Wednesday, 05:00 PM. **Thank you for your support**. Please disregard this message if you have already responded.
Nudge messaging amendments in Batch 5	[MOH] IC:01234567 You are invited to a vaccine research study on dd/mm/yy at 08:00 AM-09:00 AM. **This will measure your antibody level after vaccination**. Please respond at [survey link] by Wednesday, 5:00 PM. Thank you.	No change

The amendments to the message template are shown in bold.

A final SMS reminder was sent a day before the scheduled appointment for blood collection. A registration process was conducted at the study centre before blood collection to verify participants eligibility, explain study details and obtain written informed consent, and verify and update information collected on EVYDResearch. Blood collection was performed by trained phlebotomists. Walk in participants were accepted for blood collection as long as inclusion and exclusion criteria were fulfilled and written informed consent was obtained.

A dry run was conducted 2 weeks prior to the start of recruitment in order to test the SMS system, on-site logistics and coordination within the study team. In addition, the first batch was considered as a dress rehearsal of the workflow and response rate. Weekly meetings were held during the recruitment period to discuss the sampling population, Qualtrics response rate, number of blood samples collected and projections for response for different stratum in the following week, which provided the necessary and timely information to adapt the recruitment strategy for the next batch. Strata with low response rates were highlighted to support oversampling decisions to boost sample size for the strata. To fill up the invitation quota, priority strata were identified and allocated first. The number of invitations remaining after said allocations would be distributed equally amongst the remaining strata. Appointment days, time slots, age groups and gender strata for each vaccine brand were also balanced as much as possible. The national vaccination plans were also considered to prioritise recruitment for specific vaccine brands if required.

Serum titres for nAb (in international units per mL) were then analysed and calculated using a WHO International Standard conversion tool (30, 41). All participants NAb results were provided by SMS as positive or negative findings, and they were provided with an information sheet to help them interpret their results at the time of blood sampling (42).

## Results of the recruitment

**Figure 1:**
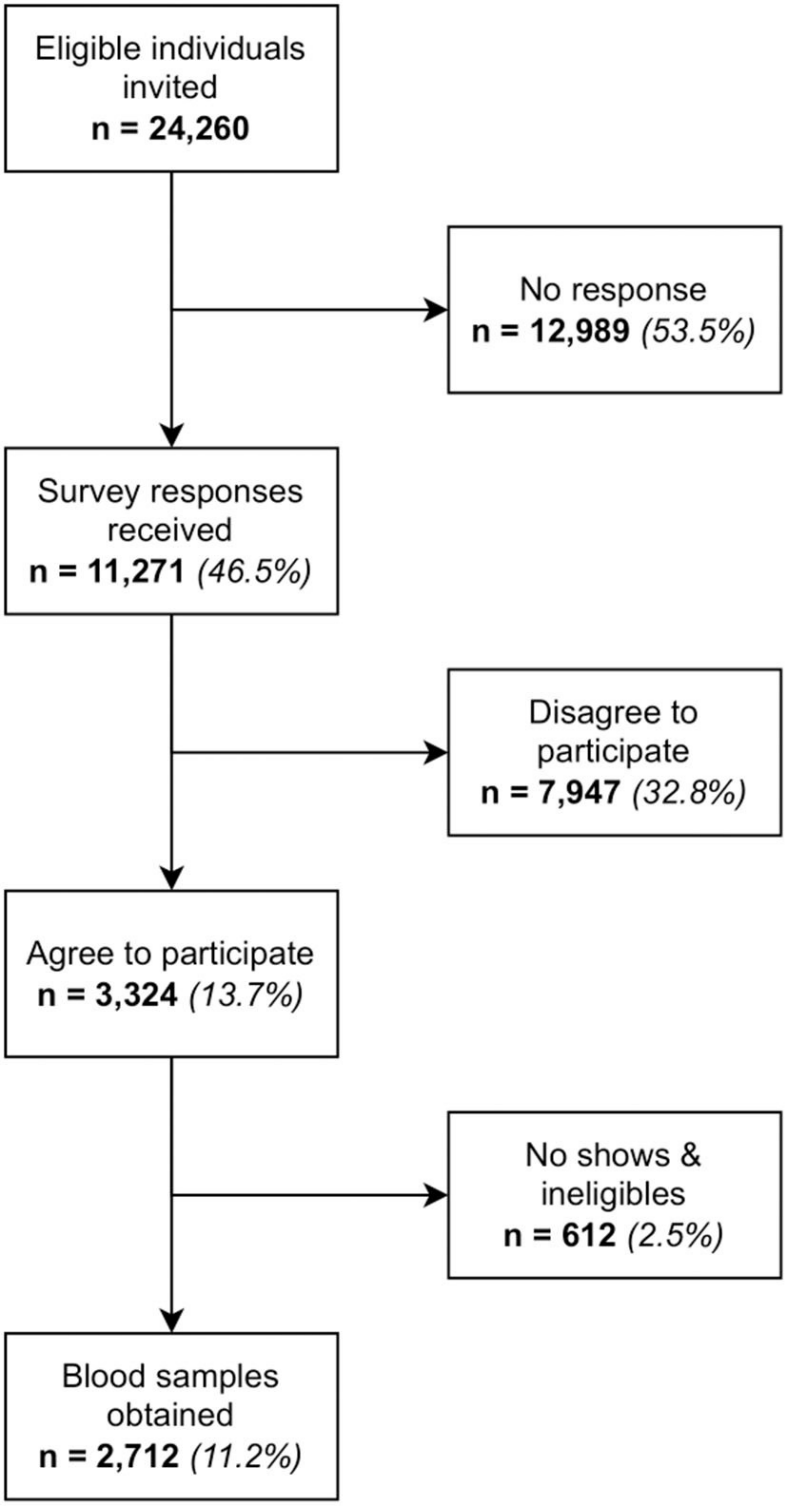
Flow diagram of recruitment numbers.

**Figure 2:**
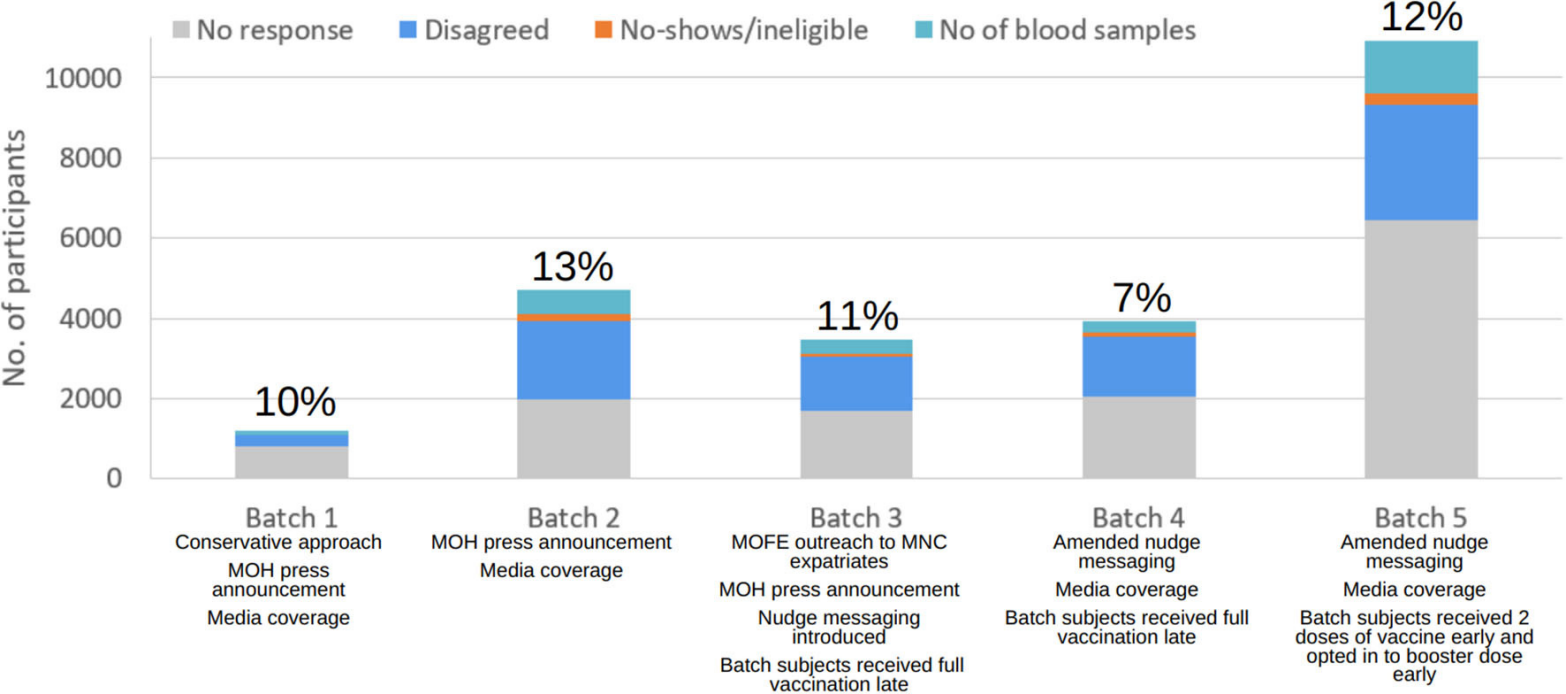
Breakdown of recruitment numbers and response rates by batch. Interventions taken and participants' behaviours in each batch are as listed and further described in sections “Results of the recruitment” and “Discussion”.

A total of 24,260 individuals were invited to the study. 2,712 blood samples were obtained, which equated to a response rate of 11.2%. Target numbers were met across all age and gender strata for MRNA-1273 but fell short for AZD1222 and BBIBP-CorV. Adjusting the invitation numbers and adjusting the recruitment strategy at each batch had helped to control the sample size of each stratum. Refer to Figures 1, 2, Supplementary Table 6, and Tables 3, 4 for further breakdown of the recruitment process. The recruitment strategy for each batch is described as below.

**Table 3:** At the end of the recruitment, blood samples were collected from a total of 2,712 participants with the below strata distribution by vaccine brand, age and gender.

	**Female**					**Male**				
	**18–30**	**31–40**	**41–50**	**51–60**	**>60**	**18–30**	**31–40**	**41–50**	**51–60**	**>60**
AZD1222 (646)	96	100	85	97	11	58	43	58	80	18
mRNA−1273 (1775)	174	185	178	244	123	150	173	175	220	153
BBIBP–CorV (291)	70	23	25	26	4	59	28	33	20	3

**Table 4:** The total distribution of the blood samples collected according to the respective strata of the number of weeks post–vaccination.

	**3 weeks**	**4 weeks**	**5 weeks**	**6 weeks**
AZD1222 (646)	44	96	175	331
mRNA−1273 (1775)	644	712	155	264
BBIBP–CorV (291)	24	13	108	146

### Batch 1

Being the first live run of the recruitment, a conservative approach was taken to avoid overstretching resources. There was media coverage and ministerial support for the initial recruitment. 1,200 people were invited into the study, and it was predicted that 60% of the invitees would turn up. When the list of eligible individuals for the current and upcoming batches was evaluated, it was noted that there was a small and limited population of those who received the BBIBP-CorV vaccine. Hence, the priority was to invite all individuals who received the BBIBP-CorV vaccine and were at post vaccination weeks 5 and 6. The remaining quota was equally distributed between AZD1222 and MRNA-1273 sampling groups who were 6 weeks post-vaccination. Based on the final response rate of 10% in Batch 1, the response rate for Batch 2 was adjusted to 20%.

### Batch 2

Like in Batch 1, all eligible individuals who received the BBIBP-CorV vaccine were prioritised. As there was a lack of BBIBP-CorV immunised population, individuals who had received a booster dose following a primary series of BBIBP-CorV at 5 to 6 weeks post-vaccination (defined as 2 BBIBP-CorV doses followed by 1 dose of MRNA-1273/BNT162b2) began to be included in the study. Due to insufficient sample size, the remaining quota was distributed evenly between AZD1222 and MRNA-1273 groups who were 6 weeks post-vaccination. The Minister of Health expressed his support for this study once again. Ultimately, we invited 4,723 subjects with a 13% response rate.

### Batch 3

The study team continued to assume a response rate of 20%. As there were only 134 eligible BBIBP-CorV individuals, including the booster group, all were invited. The AZD1222 and MRNA-1273 groups who were 4 to 5 weeks post-vaccination were then invited into the study.

The nudge text was also introduced. In addition, there was also targeted recruitment of BBIBP-CorV vaccinated expatriates from a prominent multi-national company (MNC). The vaccination centre administered booster doses to this subgroup. As the expatriates' first language is Mandarin Chinese and they could not understand English or Malay, it was possible they were unable to participate in the study due to the language barrier. To avoid losing out on these MNC expatriates as potential participants, their HR department was contacted *via* the Ministry of Finance and Economy so that the study can be communicated to them in Mandarin Chinese.

Despite targeted recruitment and nudge messaging, overall response was poorer, with only 374 blood samples obtained from the invited 3,482 people, garnering a reduced response rate of 11%.

### Batch 4

The expected response rate was revised down to 15%. As only one-third of the targeted sample size was reached, more resources were added to increase the recruitment effort. Eligible individuals who received the BBIBP-CorV primary series vaccines, and Week 5 to 6 BBIBP-CorV primary series plus booster vaccines were invited into the study. Following that, eligible individuals who received AZD1222 and MRNA-1273 primary series who were 3 to 5 weeks post-vaccination were invited. Due to smaller than expected sample size of AZD1222 and MRNA-1273, those who received either the BNT162b2 or MRNA-1273 booster doses were invited to fill up the remaining quota. Despite efforts to engage the public in the study *via* social media and nudge messaging, the response rate was the poorest at 7% with only 284 blood samples collected.

### Batch 5

As this was the last week for recruitment, the maximum capacity was further increased in an attempt to reach the targeted sample size. The predicted response rate of 15% was maintained. The current recruitment progress was reviewed and it was noted that that the age groups of over 50 years were undersampled across all vaccine brands. There was interest to investigate how immunosenescence in individuals over 50 years of age affects nAb levels (43). The age groups of 50 to 60 years, and above 60 years were oversampled by inviting all eligible individuals over 50 years old into the study, independent of the vaccine brands they had received.

As the BBIBP-CorV vaccinated group remained undersampled, all eligible individuals who had received the BBIBP-CorV vaccines were recruited into the study. Similarly, all eligible individuals who received AZD1222 and MRNA-1273 vaccines respectively were invited into the study. Females who were given the MRNA-1273 vaccines were undersampled and allocated last as they were already overrepresented in the study compared to males. The response rate for Batch 5 is 12% with 1,304 blood samples obtained from the 10,913 people who were invited.

The nudge messaging was also amended to better highlight the personal benefits of joining the study. There was also media coverage of the study during the recruitment.

## Discussion

Many studies have been abandoned because they failed to reach a sufficient sample size in a timely manner (44). Despite deploying resources across multiple centres for recruitment, it can still be difficult to achieve the desired sample size in a short amount of time (45). In multi-centred recruitment, information on the recruitment progress will need to be shared by different sites and aggregated together for discussion and alignment before implementing any changes to the recruitment strategy (46, 47). The recruitment workflow described here demonstrates a responsive and agile approach to large-scale recruitment with the use of technological solutions to successfully achieve the recruitment target in a short period of time. The below outlines and justifies the intervening strategies which improved the response rate of the study.

### Data extraction

The access to Bru-HIMS and BruHealth have facilitated the auto-generation of the full sampling frame of eligible participants who meet the inclusion and exclusion criteria. This enabled us to carry out a more accurate and rigorous stratified sampling and avoids the issues of convenience sampling. Pre-assigning appointment slots to potential participants based on the expected response rate in the invitations is an effective crowd control measure and prevents overstretching of resources at the recruitment centre. Pre-planning the schedule ensured a pleasant experience for both participants and staff which adheres to COVID-19 social distancing and venue capacity guidelines.

EVYDResearch played a key role in providing up-to-date information which influenced the recruitment strategies. It has a graphical user interface which has a dashboard that shows the overall recruitment progress according to different stratification factors in the sampling scheme by showing the live sample size of each stratum, e.g., age, gender, vaccine brands, time from latest dose to blood collection etc., (Supplementary Figure 2).

Users can conduct conditional search queries visualised on tree diagrams in EVYDResearch *via* drag and drop and drop-down lists to filter the fields and conditions based on the inclusion and exclusion criteria (Supplementary Figure 3). Coding knowledge is not required to conduct the searches. Multiple cohort searches can be run. The search queries and results can be saved as projects and can be refreshed to reflect live data. These projects can also run simple statistical analyses such as descriptive statistics and univariate analysis.

During the weekly meeting, these statistics were generated quickly so that stakeholders could see the current recruitment progress by strata breakdown. Based on this information, sampling strategies had been proposed to try and improve response rates. Using Supplementary Figure 4 as an example, the univariate analysis revealed lower than targeted numbers of females above 60 years of age who received the AZD1222 vaccine. This would mean that more individuals from this stratum would need to be invited for the following batch in order to increase the sample size.

### Recruitment by SMS

The eligible participants can be contacted directly as their contact details are available on Bru-HIMS and BruHealth. Contacting by phone can be a tedious, costly and time-consuming process, and does not guarantee a good response rate (48–51). Although emails are convenient and cost-effective, response rates are poor as they are usually ignored (52). Considering the large numbers of participants that had to be invited into the study and 88% smart phone ownership in Brunei as of 2018 (53), SMS was considered as the best communication method for recruitment especially when the messaging process could be automated. Furthermore, recruitment *via* SMS generated higher participation rates compared to emails (54). The study by Amorim et al. (55) showed the effectiveness of SMS for recruitment as well. Having MOH as the sender ID provided the government endorsement for the study and improved legitimacy. Like many providers around the world, SMS is also utilised by various governmental agencies, including MOH, to contact people for official matters like appointment confirmations. The use of SMS to send appointment reminders are effective in ensuring attendance (56).

### Strategies to improve response rate

#### Nudge messaging

Incorporating nudges into text messages is a form of applied behavioural science to encourage participation in desirable positive behaviours at low costs, such as encouraging the uptake of influenza vaccines and implementing strategic policies (57, 58). Nudges are an interventional tool to influence choices without imposing mandates or bans (59). Initially, introducing the nudge messaging of “This will measure your antibody level after vaccination” in Batch 3 did not improve response rate in Batches 3 and 4. This could be because the cohorts could be less receptive to vaccinations. This is further discussed in section human behaviour. Adding another nudge messaging of “An antibody test is reserved for you next week” in Batch 5 improved response rate to 12%. It could be inferred that the content and style of the nudge messaging play a more important role in influencing behaviour than the act of nudge alone.

#### Implementation intention prompts

Appointment slots have been pre-assigned to each participant about 2 weeks in advance to better manage the flow of people at the recruitment centre and to comply with social distancing guidelines. The inclusion of a date and a time for the blood sampling appointment was not just a logistical convenience, it also acted as a nudge to encourage autonomous behaviour towards willing participation (60).

Giving people a clear and concrete plan encourages their cooperation to attend the desired future appointment (61). Imposing a short deadline to respond to the survey creates a sense of urgency that spurs participants into completing the survey and indicate their commitment to the study (62). Having the appointment dates set for the following week encourages participation as participants only need to evaluate if they can attend the appointment (63).

#### Governmental support

To establish legitimacy of the research, offline support was sought from governmental agencies such as MOH. The Minister of Health has made press announcements about the study and how it would inform future health policies, and urged invited citizens to participate (64, 65). Organisational and professional credentials give people the confidence to trust that the research is safe, ethical and benefiting the society (66). This is especially important in Brunei where the transparent leadership shown in the handling of the COVID-19 pandemic has already cemented public trust in the government (67). When the press releases were made, the response rates climbed to as high as 13%.

The study team noted that expatriates from the People's Republic of China working in a multi-national company (MNC) attended the vaccination centre en masse to obtain their BBIBP-CorV vaccine. Vaccination staff on-site noted they are not conversant in the working languages of Brunei which are Malay and English. There was the possibility that this BBIBP-CorV sub-group could have been missed out on the study due to their inability to comprehend the SMS invitation in English and Malay. A decision was made to reach out to this group directly. Assistance was sought from the Ministry of Finance and Economy and the Brunei Investment Agency, who engaged the Human Resources team of the MNC. Internal communication of the study and purpose of recruitment was conducted in Mandarin Chinese. This direct approach allowed us to recruit an eligible sub-group who was initially out of reach due to the language barrier. Language barriers could limit responses and considerations will need to be made to strategise the communication and outreach strategies (68).

#### Enquiries hotline

SMS, patient information sheets and survey questionnaires are convenient and contained all relevant details relating to the study. As the entire recruitment process was done online, there was no opportunity for individuals to clarify any questions. A nominated WhatsApp hotline was set up by the study team to attend to enquiries. Having extended discussions with the subject experts on the study would improve individuals' understanding and increased their likelihood of consenting to participate in the study (69). Getting assistance with technical difficulties could also overcome some barriers of recruitment and improve response rate, such as inability to navigate the survey form. More than 400 texts and calls were received over the recruitment period. Feedback can be grouped into clarifications on the study, logistics and research outcomes (Table 5).

**Table 5:** Enquiries received by the project hotline.

**Nature of enquiry**	**General examples of questions received**
Clarifications on the study	• What is this study about? • Reasons why they were chosen for this study. • Scepticism towards vaccination or the study.
Logistics	• Checking if survey responses were submitted successfully. • Penalties for non–participation. • Incentives for participation. • Is this the appointment slot for the booster dose? • Is this a blood donation drive? • Location of the blood drawing. • Fasting requirements for blood sampling. • Transportation issues. • Transferring the invitation to participate to a third party. • Enrolment of interested non–eligible individuals into the study. • Volunteering opportunities for the study.
Research outcomes	• When will the results be released? • Action plan if levels of nAb is low. • Arrangements for booster dose if nAb is low.
Suggestions for improvements	• Content of SMS and patient information sheet • Incentives for participation • Blood drawing at other locations.

Feedback received from participants who contacted the enquiries hotline has refined the communication of the recruitment. For example, the lengthy patient information sheet was hosted on a separate webpage instead of the Qualtrics survey questionnaire as it caused confusion and had led to incomplete questionnaires. There were also some suggestions for improvement which could not be feasibly implemented due to limitation in logistics, such as provision of incentives and phlebotomy services at other sites. Additionally, the feedback received had also given insights on participants' behaviour and receptivity to the study which can inform the planning of future recruitment. This is further elaborated in Section Human behaviour.

By deploying the handling of enquiries to an offsite location, the recruitment centre was able to focus solely on sampling blood from confirmed participants. This allowed for fast turnover of blood sampling with minimal interruptions to onsite logistics.

#### Media coverage

One of the barriers to recruitment is the lack of awareness of the studies being conducted (13, 70). Media campaigns could help to increase public awareness in research participation (71). To ensure the different age demographics were reached, the study was covered by a variety of media outlets including print media, television and social media. In Brunei, the younger population are more frequent users of social media while the older population tend to consume traditional media (72, 73). Traditional media also cross-posted their coverage on the study recruitment on their respective social media accounts. As the general population in Brunei have a high level of trust in the local state media (74), having media coverage of the study in multiple outlets could have had an influential role in study participation.

There was traditional media coverage during early stages of the recruitment to encourage participation, including a televised interview with the study team who explained the purpose of the study to increase public understanding (64, 65, 75).

In Brunei, there is a heavy usage of social media amongst most of the population that are smartphone dependent (72). Hence, social media was a suitable platform to spread awareness and understanding of the study to the masses especially the younger population, hence indirectly increasing response rates. MOH and the study team have published social media posts and videos on their Instagram accounts, with high public engagement by responding to comments (76–79). The higher response rates in Batch 1, 2 and 5 coincided with the publication of social media posts during the invitation week of said batches. The timing and consistency of the social media posting might have played a role in influencing response rates as response rates were lower in Batches 3 and 4 when there was lesser media coverage of the recruitment.

### Limitations

#### Delays to recruitment

Time taken for funding and ethics approval would need to be considered and proactively buffered for in the planning stages as the start of recruitment could be delayed and even impact the sample size, especially when the inclusion criteria are time-sensitive (80–82). Although ample preparations have been made, the study was delayed by a few months as the sudden introduction of government-mandated restricted movements in Brunei had halted most research operations when the second wave of the COVID-19 pandemic hit Brunei in August 2021.

Based on the National COVID-19 Immunisation Programme, BBIBP-CorV, AZD1222 and MRNA-1273 COVID-19 vaccines were rolled out in the order of their procurement (83–86). When recruitment finally commenced, the population of BBIBP-CorV vaccinated individuals who were at 2–6 weeks after vaccination was small as most of the population received AZD1222 and MRNA-1273 vaccines by default (83). As part of the adaptive recruitment strategy to maintain adequate power, the inclusion criteria were modified to include booster populations into the study.

#### Human behaviour

The recruitment team noted a spectrum of education levels and health literacy amongst the participants. Based on some of the enquiries received in Table 5, some participants confused the study for a booster vaccination appointment or a blood donation drive. Others thought the SMS they received was a scam. Successful recruitment requires addressing and considering participants' level of understanding so that they are capable to give informed consent (69). The enquiries hotline, government endorsement and media coverage played important roles in educating, clarifying doubts and instilling confidence in participants to take part in this study. The linking of the purpose of the study to informing health policies probably motivated some to enrol into the study as an act of patriotism or altruism (87). The disclosure of participants' nAb levels *via* SMS, together with the support of medical doctors manning the enquiries hotline to explain what the results mean, promote transparency of the study with tangible results benefitting the participants.

Despite the various strategies to encourage recruitment, it seemed that participants' personal views on COVID-19 vaccinations influenced their receptivity to participate in the study. For context, Brunei entered the transition phase of the National COVID-19 Recovery Framework on 19 November 2021 when 70% of its population had received two doses of COVID-19 vaccine (88). All public-facing employees must have received their full vaccination, and patrons to public amenities must be fully vaccinated (89). The announcement of the framework could mean that many anti-vaccinationists might feel coerced to become vaccinated in November so that they can partake in their usual social activities during the transition phase. By the time they received their full vaccination in December, they could have been invited to participate in Batch 3 or 4 as they meet the eligibility criteria. They were likely to express similar reservations towards research studies on COVID-19 vaccines, and this was reflected by an increase in hostile comments about the study and the vaccine by the enquiries hotline. This could be the main reason for the lower-than-expected response rates in batches 3 and 4 despite the implementation of nudge messaging and earlier media coverage.

The behaviour of Batch 5 was a stark contrast to Batches 3 and 4. A greater proportion of Batch 5 participants were recipients of booster doses who received their vaccinations in mid-December when MOH expedited the administration of booster doses in light of the developing Omicron variant (90). Participants who received their booster shots demonstrated more cooperation and interest in the study as noted by the increased positive reception reported by the enquiries hotline. Based on this experience, there may be a need to consider participants' inherent beliefs towards aspects of the study when planning the recruitment strategy.

### Future operational streamlining

This study serves as a proof-of-concept study that optimal efficiency in research studies can be achieved by adaptive study recruitment and real time information sharing *via* data intelligent technology, which allows data integration and governance from multiple sources including EMR and other user-friendly frontend applications.

#### EVYDENCE data management ecosystem

EVYDENCE is the underlying data operating platform from which EVYDResearch obtained participants' RWD from. At the time of this study, EVYDENCE was still in its final stages of development and could not be fully leveraged. In the future state, EVYDENCE will form the underlying engine on which the various technological solutions are built on, creating a highly interconnected data ecosystem. With information from BruHIMS as the base, data from datasets like Qualtrics and BruHealth can also be fed into EVYDENCE. EVYDResearch will be able to extract only the necessary data from EVYDENCE in order to generate new data insights which will feedback into EVYDENCE and other solutions to form a close loop. Strict data security protocols are applied universally across the entire ecosystem to protect data confidentiality.

#### Recruitment via BruHealth

BruHealth has a bespoke backend platform, content management system (CMS) module which can be used to conduct survey-based research activities. In January 2021, a COVID-19 vaccine intention survey was rolled out on BruHealth to the public which garnered 50,658 responses (12% contact rate) (91). The mandate by MOH to check in to premises using BruHealth combined with the high average daily usage of BruHealth mean that this app can be potentially used to send push notifications to identified users to participate in this study. The survey could also be housed on BruHealth instead of a third-party server. Unfortunately, the CMS module is still in the development stages and will only be available in the first half of 2022. If CMS were to be utilised, the app can identify non-responders and push notifications to remind them to complete the submission, resulting in a more targeted notification push. This feature, once fully ready, can be used as a formal recruitment platform instead of employing an external third-party survey application. This is possible as the BruHealth app has an average of more than 300,000 daily active users as they use it for COVID-19 surveillance purposes. Participants meeting the inclusion and exclusion criteria can be identified through a connected system, and then be pushed notifications to take part in the study. Reminder notifications can also be pushed to these patients at defined intervals if they still have not responded to take part in the study, and they can be customised by researchers and incorporate various nudge techniques to get them to participate. Push notifications to targeted groups at the right times could influence participation, similar to its application in influenza vaccine uptake (92).

Invited population can indicate their interest to participate in the research study through the app and fill in relevant screening questionnaires in BruHealth, and these data can be reflected in EVYDResearch *via* EVYDENCE for data analysis. Additionally, BruHealth could incorporate an option in the user's profile to indicate an interest to participate in any research study and to be notified when a study is open for recruitment. This information could further be used to target eligible participants, which may improve the participants' response rate to join the study. Participants involved in this study could even be tagged in BruHealth so that they could be contacted to join future studies.

### Applications and future directions

This first-hand experience is proof of concept that large-scale recruitment can be done using technology and EHR. However, it is not just technology, it involves a very good planning of the workflow and understanding the requirements of the research, whilst also leveraging technology to be able to quickly plan recruitment strategies which are flexible and responsive to current recruitment progress. Being able to automate recruitment frees up manpower to focus on more vital tasks such as the data analysis and management of recruitment.

Although this proof of concept is applied to an epidemiological study, there is great potential for this recruitment methodology to be repurposed for research in other domains, especially clinical trials. With access to an EHR or a similar system, the recruitment strategy discussed can be replicated with ease. Following this methodology overcomes the limitations of traditional recruitment methods, especially by consolidating resources to one platform, automation and freeing up more bandwidth for researchers to focus on conducting the study on volunteers rather than burdening themselves with recruitment.

## Data availability statement

The original contributions presented in the study are included in the article/Supplementary material, further inquiries can be directed to the corresponding author.

## Ethics statement

The studies involving human participants were reviewed and approved by Medical and Ethical Research Committee (MHREC) of the Ministry of Health, Brunei Darussalam. The patients/participants provided their written informed consent to participate in this study.

## Author contributions

CS, SC, YWe, HG, LA, HS, MA, SH, ST, CT, XO, L-FW, YWa, AL, HL, JW, LN, and AC have contributed to the study design, study conceptualisation, and data analyses. CS drafted the manuscript in full. YWe and AC provided critical revision of the manuscript. CS, SC, YWe, YWa, AL, and HL managed data integration from BruHIMS and BruHealth. UBD and EVYD colleagues, including CS, SC, YWe, HG, LA, HS, YWa, AL, LN, and AC managed recruitment of study participants, data coordination and blood withdrawal. MA, SH, ST, and JW managed press release and policy support for the study. CT, XO, and L-FW managed nAb measurement. All authors contributed to the article and approved the submitted version.

## Funding

This research was funded by the Council for Research and the Advancement of Technology and Science (CREATES) (grant no: MITC/CREATES PROJECT UBD 001), Ministry of Transport and Infocommunications, Brunei Darussalam (Brunei Side) and Temasek Foundation (Singapore side) (grant no.: 2021-TF-0001).

## Conflict of interest

Author CS is a part-time employee of EVYD Technology Limited which was the technology partner and research collaborator of this study. EVYD Technology Limited is the developer of EVYDResearch, BruHealth and EVYDENCE platforms. SC, YWe, YWa, AL, and HL are full-time employees of EVYD Technology Limited. The remaining authors declare that the research was conducted in the absence of any commercial or financial relationships that could be construed as a potential conflict of interest.

## Publisher's note

All claims expressed in this article are solely those of the authors and do not necessarily represent those of their affiliated organizations, or those of the publisher, the editors and the reviewers. Any product that may be evaluated in this article, or claim that may be made by its manufacturer, is not guaranteed or endorsed by the publisher.
