# Supplementary material for: Technology-assisted adaptive recruitment strategy for a large nation-wide COVID-19 vaccine immunogenicity study in Brunei
Source: Front Public Health. 2022 Sep 12;10:983571. doi: 10.3389/fpubh.2022.983571 (PMC9511035; doi:10.3389/fpubh.2022.983571)
Supplement: Supplementary file 1 [file Data_Sheet_1.docx]

Supplementary Material

# Supplementary Tables

| **Fields** | **Source** | **Comments** |
| --- | --- | --- |
| Patient ID | Auto-generated | Auto-generated once invitation SMS is sent out to subjects. Used as the identifier. |
| Batch ID | Auto-generated | Batch 1, 2, 3, 4 or 5 |
| Cpass ID | Data logger | 3,000 sampling tubes are given cPass ID numbering from 1 to 3,000. Each data logger collects one sampling tube from the master rack and inputs the cPass ID into EVYDResearch prior to blood sampling. |
| Appointment Date | Auto-generated |  |
| Appointment Time Slot | Auto-generated |  |
| Age | Bru-HIMS |  |
| Age group | Auto-generated | Each subject will be automatically categorised to the respective age groups of 18-30, 31-40, 41-50, 51-60 and above 60 years old. |
| Gender | Bru-HIMS | Based on birth sex. There are only 2 possible genders recognised in Brunei, i.e. male and female |
| Telephone number | Bru-HIMS BruHealth |  |
| First vaccine dose received (brand) | BruHealth | 1st and 2nd doses are the same brand |
| First vaccine dose received (date and time) | BruHealth |  |
| Second vaccine dose received (brand) | BruHealth | 1st and 2nd doses are the same brand |
| Second vaccine dose received (date and time) | BruHealth |  |
| No. of days between the first dose and the second dose | Auto-calculated | 1. AZ = 5 weeks to 9 weeks (35 days to 63 days) 2. Moderna = 3 weeks to 8 weeks (21 days to 56 days) 3. Sinopharm = 3 weeks to 5 weeks (21 days to 35 days) |
| Third vaccine dose received (brand) | BruHealth | The third dose is either Pfizer or Moderna |
| Third vaccine dose received (date and time) | BruHealth |  |
| No. of days between 2nd/3rd dose and appointment date | Auto-calculated |  |
| Weeks post-vaccine | Auto-calculated | Based on the above no. of days between their 2nd/3rd dose and appointment date, each subject will be automatically categorised to 3, 4, 5 or 6 weeks after their second/third dose as follows: 15-21 days = 3 weeks 22-28 days = 4 weeks 29-35 days = 5 weeks 36-42 days = 6 weeks |
| Diabetes mellitus (Y/N) | Bru-HIMS | Includes all diseases listed in E10 and E11 ICD-10 codes |
| Chronic kidney disease (Y/N) | Bru-HIMS | Includes all diseases listed in N18 ICD-10 code |
| Hypertension (Y/N) | Bru-HIMS | Includes all diseases listed in I10 to I15 ICD-10 codes |
| Ischaemic heart diseases (Y/N) | Bru-HIMS | Includes all diseases listed in I20 to I25 ICD-10 codes |
| Immunosuppressive treatments (Y/N) | Bru-HIMS | Y43.3 ICD-10 code only |
| Cancers (Y/N) | Bru-HIMS | Includes all diseases listed in C00 to C97 ICD-10 codes |
| Travel history (Y/N) | Department of Immigration and National Registration | Queried based on passport number (if subject is a passport holder). To be confirmed again by data loggers before the blood draw. |
| Past COVID-19 infection (Y/N) | Bru-HIMS | U07.1 ICD-10 code only. To be confirmed again by data loggers before the blood draw. |
| Date of Qualtrics submission | Qualtrics | Only subjects who agreed will have their IC number captured and matched to existing database. |
| Ethnic Group | Qualtrics |  |
| District | Data logger |  |
| Information verified by data logger (Y/N)? | Data logger |  |
| Consent form signed? (Y/N) | Data logger |  |
| Consent given for other serology projects? (Y/N) | Data logger |  |
| Blood sampled? (Y/N) | Data logger |  |
| Time of blood draw | Data logger |  |
| Drug types | Data logger | Categorised as: 0 - No drug history 1 - Immunosuppressants 2 - Other drugs 3 - Both immunosuppressants and other drugs |
| Drug names | Data logger |  |
| Phlebotomist in charge | Data logger |  |
| Data logger in charge | Data logger |  |
| Dilution factor of serum for cPass | cPass analyst |  |
| Raw OD/absorbance value | cPass analyst |  |
| Percentage inhibition (%) | cPass analyst |  |
| Quantity of nAb (IU/ml) | cPass analyst |  |
| cPass analyst in charge | cPass analyst |  |

**Supplementary Table 1.** Metadata included in the EVYDResearch platform which captured the necessary data variables. The original data sources are listed with some further description given.

|  | Female | | | | | Male | | | | |
| --- | --- | --- | --- | --- | --- | --- | --- | --- | --- | --- |
|  | 18-30 | 31-40 | 41-50 | 51-60 | >60 | 18-30 | 31-40 | 41-50 | 51-60 | >60 |
| AZD1222  (1000) | 100 | 100 | 100 | 100 | 100 | 100 | 100 | 100 | 100 | 100 |
| mRNA-1273  (1000) | 100 | 100 | 100 | 100 | 100 | 100 | 100 | 100 | 100 | 100 |
| BBIBP-CorV  (1000) | 100 | 100 | 100 | 100 | 100 | 100 | 100 | 100 | 100 | 100 |

**Supplementary Table 2.** Targeted sample size for each stratum stratified by vaccine brand, age and gender.

|  | 3 weeks | 4 weeks | 5 weeks | 6 weeks |
| --- | --- | --- | --- | --- |
| AZD1222  (1000) | 250 | 250 | 250 | 250 |
| mRNA-1273  (1000) | 250 | 250 | 250 | 250 |
| BBIBP-CorV  (1000) | 250 | 250 | 250 | 250 |

**Supplementary Table 3.** Targeted sample size for each stratum stratified by vaccine brand and number of weeks post vaccination.

|  | **No. of weeks post vaccination** | **Total no. of participants** |
| --- | --- | --- |
| **Batch 1** | 6 weeks | 750 |
| **Batch 2** | 5 weeks | 750 |
| **Batch 3** | 4 weeks | 750 |
| **Batch 4** | 3 weeks | 750 |
| **Batch 5** | Buffer | |

**Supplementary Table 4**. The ideal number of participants to be recruited in each weekly batch. Each batch consisted of participants who had all received their vaccines during the same week. The distribution of participants across vaccine brands, age and gender are broken down in Supplementary Table 5.

|  | Female | | | | | Male | | | | |
| --- | --- | --- | --- | --- | --- | --- | --- | --- | --- | --- |
|  | 18-30 | 31-40 | 41-50 | 51-60 | >60 | 18-30 | 31-40 | 41-50 | 51-60 | >60 |
| AZD1222  (250) | 25 | 25 | 25 | 25 | 25 | 25 | 25 | 25 | 25 | 25 |
| mRNA-1273  (250) | 25 | 25 | 25 | 25 | 25 | 25 | 25 | 25 | 25 | 25 |
| BBIBP-CorV  (250) | 25 | 25 | 25 | 25 | 25 | 25 | 25 | 25 | 25 | 25 |

**Supplementary Table 5.** The ideal number of participants to be recruited for each batch stratified by vaccine brands, gender and age. The table shows even distribution across all strata. Participants for each batch had all received their vaccines during the same week as outlined in Supplementary Table 4.

|  | **Maximum capacity** | **Predicted response rate** | **Invited** | | | | **Blood sampled** | | | | **Contact rate** | **Response rate** | **Cooperation rate** |
| --- | --- | --- | --- | --- | --- | --- | --- | --- | --- | --- | --- | --- | --- |
|  |  |  | **AZD1222** | **mRNA-1273** | **BBIBP-CorV** | **Total** | **AZD1222** | **mRNA-1273** | **BBIBP-CorV** | **Total** |  |  |  |
| **Batch 1** | 750 | 60% | 201 | 210 | 789 | 1,200 | 14 | 21 | 84 | 119 | 32% | 10% | 10% |
| **Batch 2** | 750 | 20% | 1913 | 2110 | 700 | 4,723 | 277 | 224 | 130 | 631 | 58% | 16% | 13% |
| **Batch 3** | 700 | 20% | 1663 | 1,685 | 134 | 3,482 | 211 | 129 | 34 | 374 | 52% | 13% | 11% |
| **Batch 4** | 980 | 15% | 706 | 2,966 | 270 | 3,942 | 63 | 191 | 30 | 284 | 48% | 10% | 7% |
| **Batch 5** | 1155 | 15% | 946 | 9,684 | 283 | 10,913 | 81 | 1210 | 13 | 1,304 | 41% | 14% | 12% |

**Supplementary Table 6.** Final recruitment breakdown by strata. Contact rate = Percentage of invitees who submitted their response on Qualtrics. Response rate = Percentage of invitees who agreed to participate in the study. Cooperation rate = Percentage of individuals who actually showed up on the day to donate their blood sample given.

# Supplementary Figure Caption

**Supplementary Figure 1.** Recruitment workflow.

**Supplementary Figure 2.** Example dashboard showing the overall profiles of the participants.

**Supplementary Figure 3.** Conditional tree search on EVYDResearch which allows users to visualise their searches and see the yield of the search results.

**Supplementary Figure 4.** Univariate analysis conducted on EVYDResearch. The example analysis shows the strata breakdown for the AZD1222-vaccinated participants.
