# Supplementary figures and images for: Technology-assisted adaptive recruitment strategy for a large nation-wide COVID-19 vaccine immunogenicity study in Brunei
Source: Front Public Health. 2022 Sep 12;10:983571. doi: 10.3389/fpubh.2022.983571 (PMC9511035; doi:10.3389/fpubh.2022.983571)

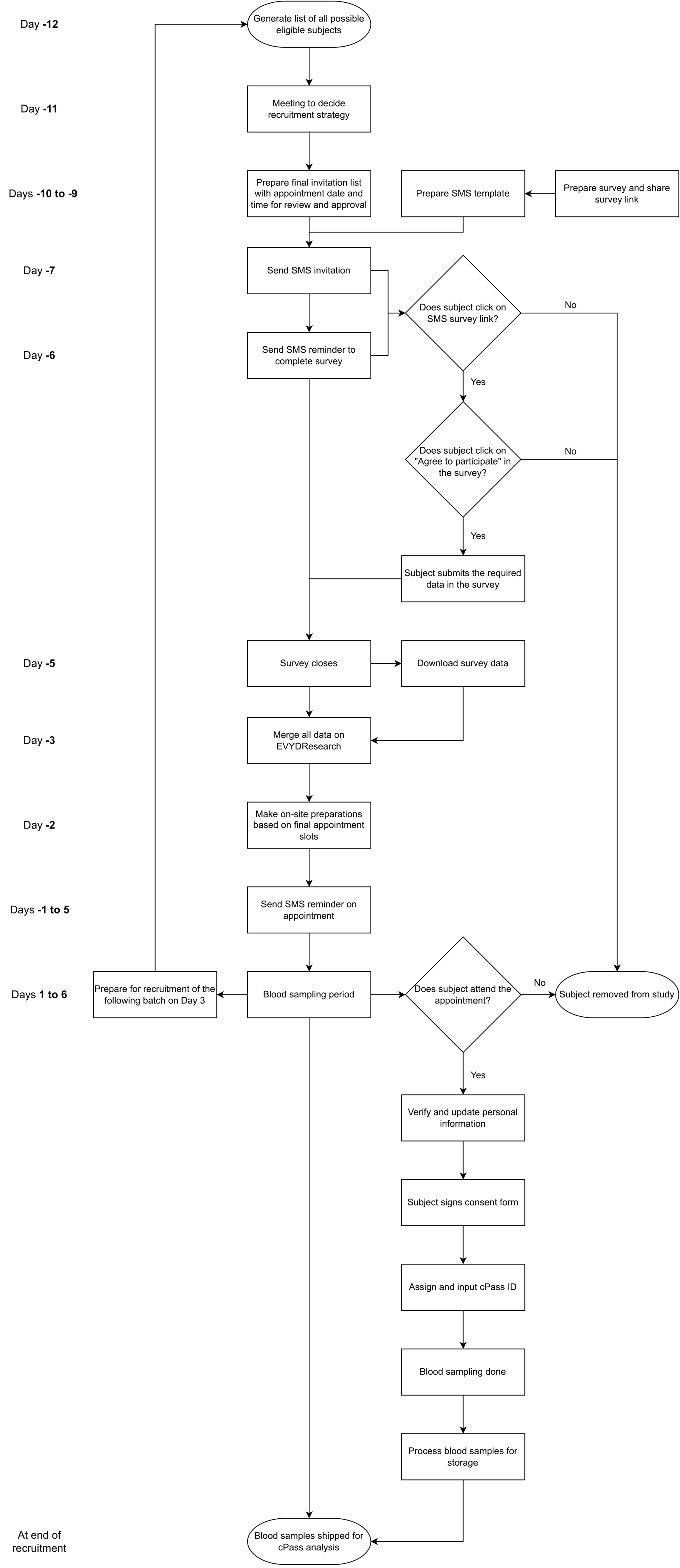

Supplement: Supplementary file 2 [file Image_1.TIFF]

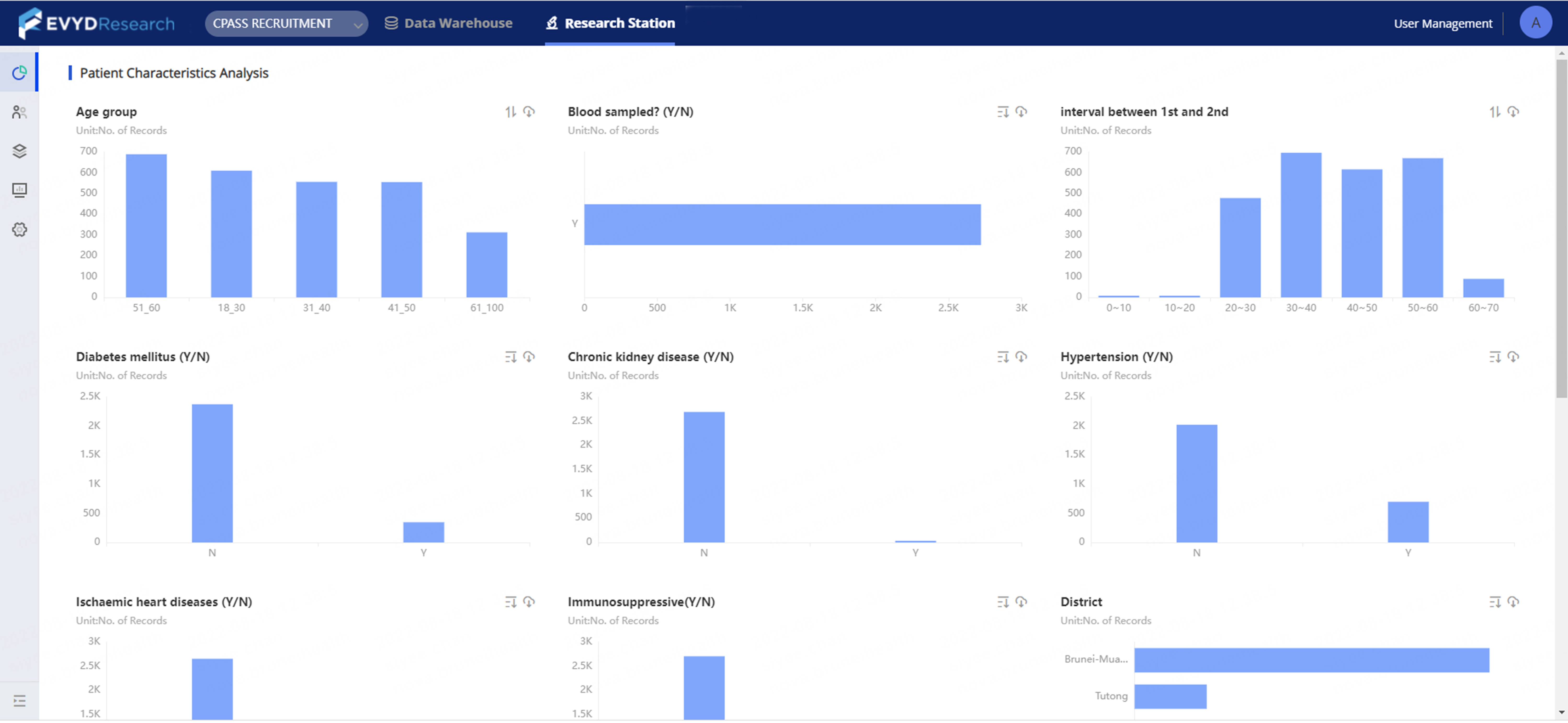

Supplement: Supplementary file 3 [file Image_2.TIFF]

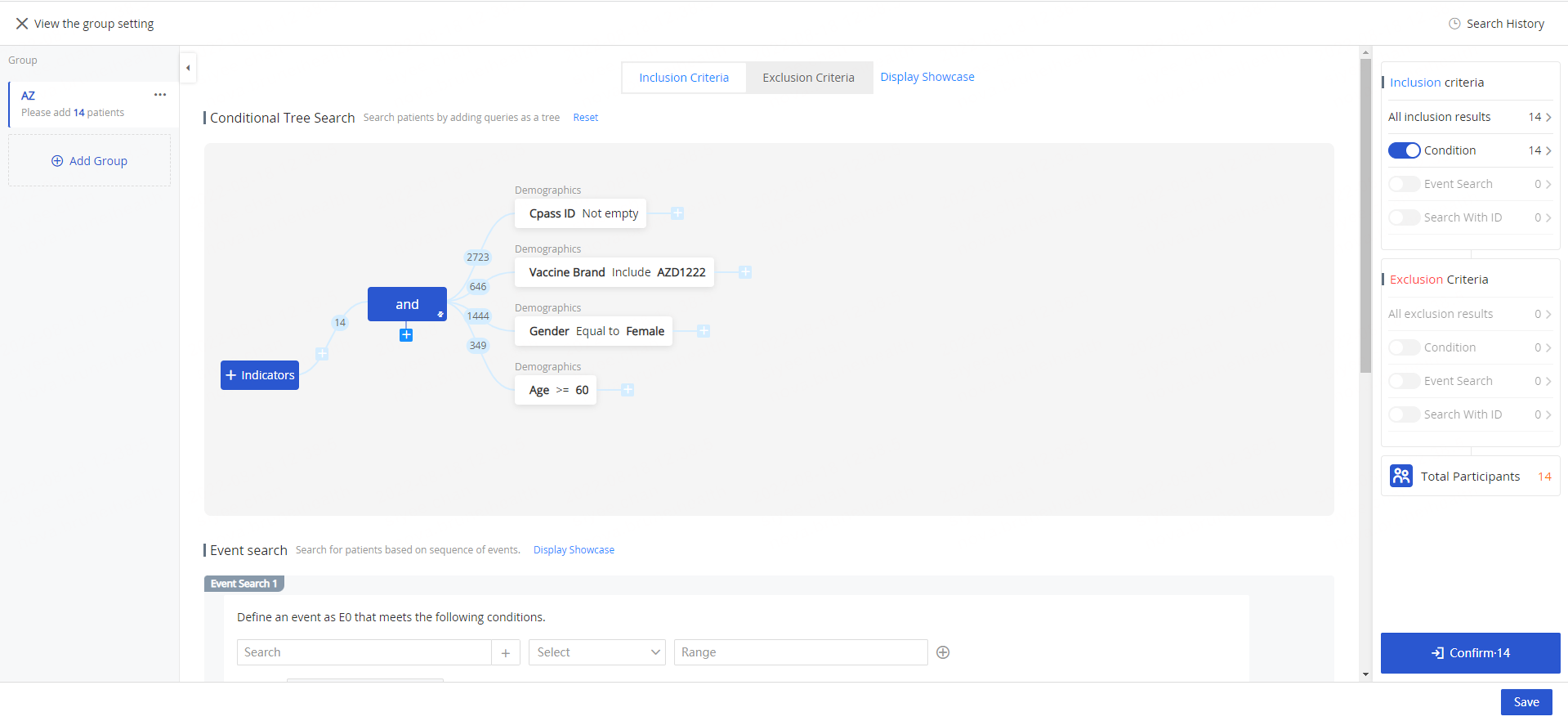

Supplement: Supplementary file 4 [file Image_3.TIF]

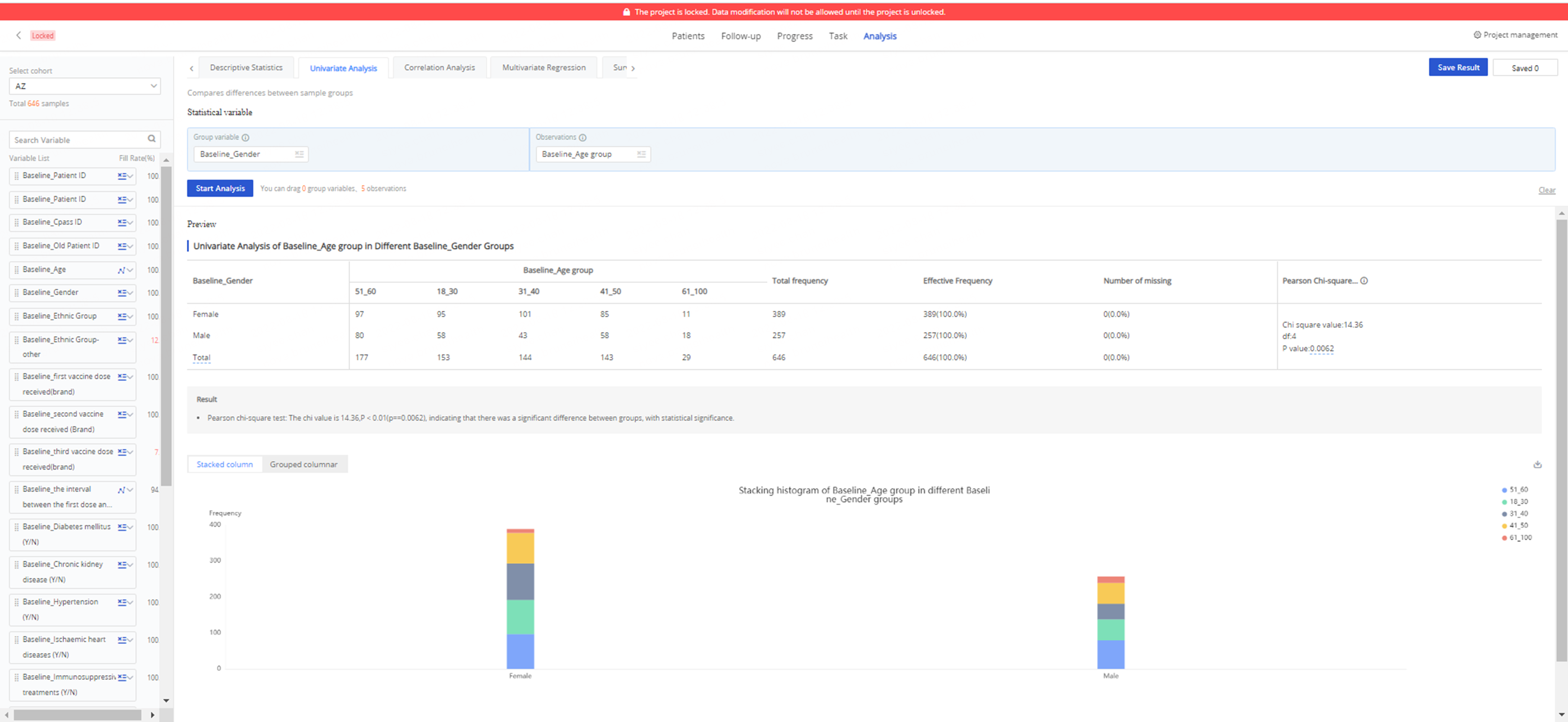

Supplement: Supplementary file 5 [file Image_4.TIF]
